# Supplementary material for: Causal Relationship between Aging and Anorexia Nervosa: A White-Matter-Microstructure-Mediated Mendelian Randomization Analysis
Source: Biomedicines. 2024 Aug 16;12(8):1874. doi: 10.3390/biomedicines12081874 (PMC11351342; doi:10.3390/biomedicines12081874)
Supplement: Supplementary file 1 [file biomedicines-12-01874-s001.zip › Supplementary Figure/Figure S4/Singe SNP plot of MR analysis for TL to ubm-b-1696.pdf]

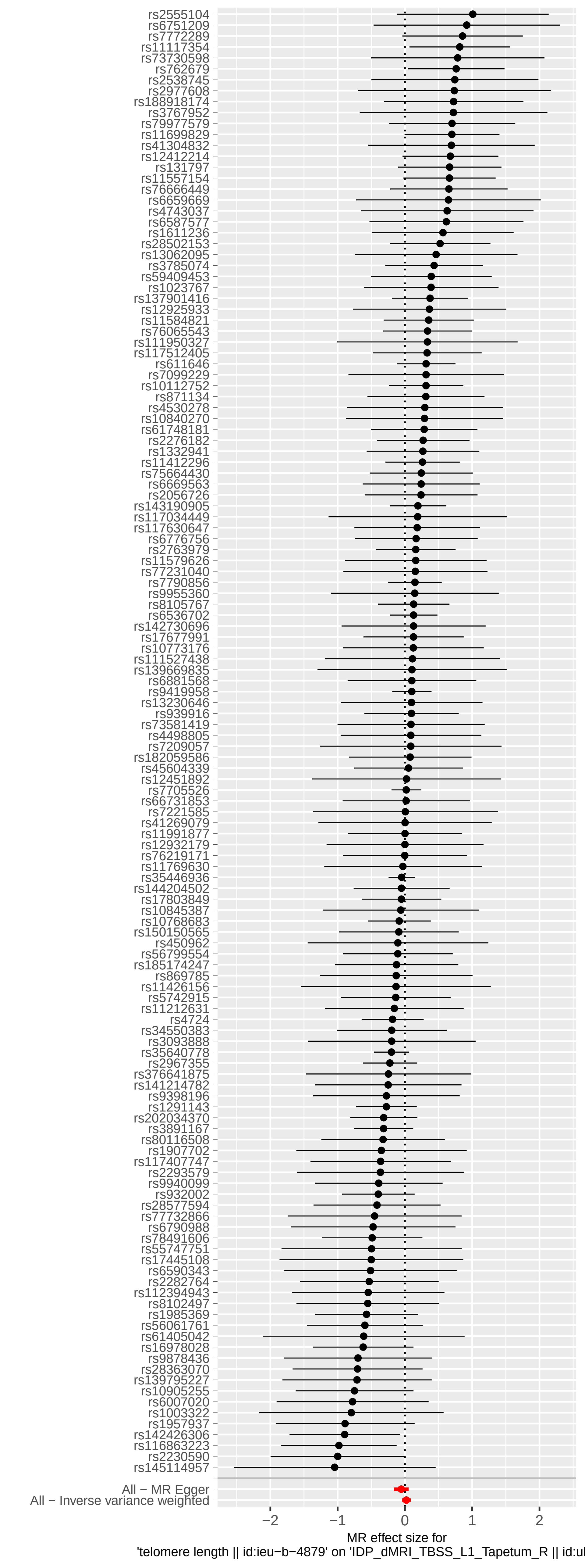
rs2555104
rs6751209
rs7772289
rs11117354
rs73730598
rs762679
rs2538745
rs2977608
rs188918174
rs3767952
rs79977579
rs11699829
rs41304832
rs12412214
rs131797
rs11557154
rs76666449
rs6659669
rs4743037
rs6587577
rs1611236
rs28502153
rs13062095
rs3785074
rs59409453
rs1023767
rs137901416
rs12925933
rs11584821
rs76065543
rs111950327
rs117512405
rs611646
rs7099229
rs10112752
rs871134
rs4530278
rs10840270
rs61748181
rs2276182
rs1332941
rs11412296
rs75664430
rs6669563
rs2056726
rs143190905
rs117034449
rs117630647
rs6776756
rs2763979
rs11579626
rs77231040
rs7790856
rs9955360
rs8105767
rs6536702
rs142730696
rs17677991
rs10773176
rs111527438
rs139669835
rs6881568
rs9419958
rs13230646
rs939916
rs73581419
rs4498805
rs7209057
rs182059586
rs45604339
rs12451892
rs7705526
rs66731853
rs7221585
rs41269079
rs11991877
rs12932179
rs76219171
rs11769630
rs35446936
rs144204502
rs17803849
rs10845387
rs10768683
rs150150565
rs450962
rs56799554
rs185174247
rs869785
rs11426156
rs5742915
rs11212631
rs4724
rs34550383
rs3093888
rs35640778
rs2967355
rs376641875
rs141214782
rs9398196
rs1291143
rs202034370
rs3891167
rs80116508
rs1907702
rs117407747
rs2293579
rs9940099
rs932002
rs28577594
rs77732866
rs6790988
rs78491606
rs55747751
rs17445108
rs6590343
rs2282764
rs112394943
rs8102497
rs1985369
rs56061761
rs61405042
rs16978028
rs9878436
rs28363070
rs139795227
rs10905255
rs6007020
rs1003322
rs1957937
rs142426306
rs116863223
rs2230590
rs145114957
All − MR Egger
All − Inverse variance weighted
−2
−1
0
1
2
MR effect size for
'telomere length || id:ieu−b−4879' on 'IDP_dMRI_TBSS_L1_Tapetum_R || id:ul
